# Circ003429 Regulates Unsaturated Fatty Acid Synthesis in the Dairy Goat Mammary Gland by Interacting with miR-199a-3p, Targeting the *YAP1* Gene

**DOI:** 10.3390/ijms23074068

**Published:** 2022-04-06

**Authors:** Peixin Jiao, Meimei Zhang, Ziwei Wang, Gege Liang, Xiaolai Xie, Yonggen Zhang, Zhi Chen, Qianming Jiang, Juan J. Loor

**Affiliations:** 1 College of Animal Science and Technology, Northeast Agricultural University, Harbin 150030, China; jiaopeixin@163.com (P.J.); 15546120650@163.com (M.Z.); wzw17390606858@163.com (Z.W.); 13422166268@163.com (G.L.); xlxie123@126.com (X.X.); zhangyonggen@sina.com (Y.Z.); 2 Jiangsu Key Laboratory of Animal Genetic Breeding and Molecular Design, College of Animal Science and Technology, Yangzhou University, Yangzhou 225009, China; 3 Mammalian Nutrition Physiology Genomics, Department of Animal Sciences and Division of Nutritional Sciences, University of Illinois, Urbana, IL 61801, USA; qj5@illinois.edu

**Keywords:** circ003429, fatty acid synthesis, goat mammary epithelial cells, miR-199a-3p, *YAP1*

## Abstract

Fatty acid composition is a key factor affecting the flavor and quality of goat milk. CircRNAs are now recognized as important regulators of transcription, and they play an important role in the control of fatty acid synthesis. Thus, understanding the regulatory mechanisms controlling this process in ruminant mammary glands is of great significance. In the present study, mammary tissue from dairy goats during early lactation and the dry period (nonlactating) were collected and used for high-throughput sequencing. Compared to levels during the dry period, the expression level of circ003429 during early lactation was lower (12.68-fold downregulated). In isolated goat mammary epithelial cells, circ003429 inhibited the synthesis of triglycerides (TAG) and decreased the content of unsaturated fatty acids (C16:1, C18:1, and C18:2), indicating that this circRNA plays an important role in regulating lipid synthesis. A binding site for miR-199a-3p in the circ003429 sequence was detected, and a dual-luciferase reporter system revealed that circ003429 targets miR-199a-3p. Overexpression of circ003429 (pcDNA-circ003429) downregulated the abundance of miR-199a-3p. In contrast, overexpression of miR-199a-3p increased TAG content and decreased mRNA abundance of Yes-associated protein 1 (*YAP1*) (a target gene of miR-199a-3p), and TAG content was decreased and mRNA abundance was increased in response to overexpression of circ003429. These results indicate that circ003429 alleviates the inhibitory effect of miR-199a-3p on the mRNA abundance of *YAP1* by binding miR-199a-3p, resulting in subsequent regulation of the synthesis of TAG and unsaturated fatty acids.

## 1. Introduction

The mammary gland is the most important lipid-synthesizing organ during lactation [1]. Regardless of species, the lipids in milk are composed of 98% triglycerides (TAG) and a small percentage of phospholipids and sterols [2]. Triglycerides are synthesized using fatty acids and glycerol as raw materials in the endoplasmic reticulum, and they are then secreted into the cytoplasm, where they fuse with one another, gradually increasing in size, and are then enveloped with specific proteins to form lipid droplets [3]. The assembled lipid droplets of different sizes pass through the cytoplasm to reach the cell membrane, where they merge and are then secreted to form milk fat droplets [4]. There are two primary sources of fatty acids in milk: one is synthesized de novo in mammary epithelial cells, while the other is derived from absorbed fatty acids from the blood [5]. Both sources are used in the assembly of lipid droplets in mammary cells prior to their secretion into milk.

Historically, the goat has been an important species in which various aspects of physiological, endocrine, and molecular regulation of milk fat synthesis have been studied. Among recent findings using goat mammary epithelial cells (GMECs), the recognition that large tumor suppressor kinase 1 (*LATS1*) inhibits the abundance of the transcription regulator Yes-associated protein 1 (*YAP1*) is particularly important from a mechanistic standpoint. This protein is a downstream component of the Hippo signaling pathway that plays a vital role in a number of cellular functions [6]. Overexpression of *YAP1* inhibits the formation of lipid droplets under the action of SOX2, thus underscoring its biological role in lipid synthesis [7]. The insulin-sensitive kinase, *IRS2*, is a target gene of miR-181b, which also promotes the expression of *YAP1* [8], highlighting a novel role of this miRNA not only on in regulation of the Hippo signaling pathway but also in the synthesis of TAG and ultimately the level of milk fat. Despite these observations, mechanisms whereby *YAP1* exerts its function on lipid synthesis in GMECs are unclear. Further exploration of the mechanism of *YAP1* and its upstream regulatory mechanism is of great significance for determining the regulatory mechanism that controls milk fat in dairy goats.

Noncoding RNAs (ncRNAs) control important physiological aspects across a number of organisms [9,10]. Most circular RNAs (circRNAs) are ncRNAs primarily derived from exons; the same genetic locus may produce multiple circRNAs through selective cyclization that are stable and not easily degraded, underscoring their important role in a number of biological functions [11,12]. Although work on circRNAs in livestock species is in its infancy, recent studies have uncovered important biological functions for these molecules, e.g., regulation of myoblast development and milk fat metabolism in bovines [13,14]. Most circRNAs have multiple microRNA (miRNA)-binding sites and competitively bind miRNAs to regulate target genes adsorbed by miRNAs [15]. It has been reported that circ01592 inhibits the function of miR-218, and circHIPK3 inhibits the function of miR-124-3p [14,16]. Moreover, we found 215 differentially expressed circRNAs (including circ003429) in goat mammary gland tissues between the early and dry/nonpregnant stages, indicating a potential effect of circRNAs on fatty acid synthesis. Given these findings, we hypothesized that circRNAs regulate goat mammary fatty acid synthesis by competitively inhibiting miRNAs. Thus, the main objective of this study was to investigate the biological function of circ003429 along with its potential role in regulating fatty acid synthesis.

## 2. Results

### 2.1. High-Throughput Sequencing of Mammary Gland Tissue

**Figure 1:**
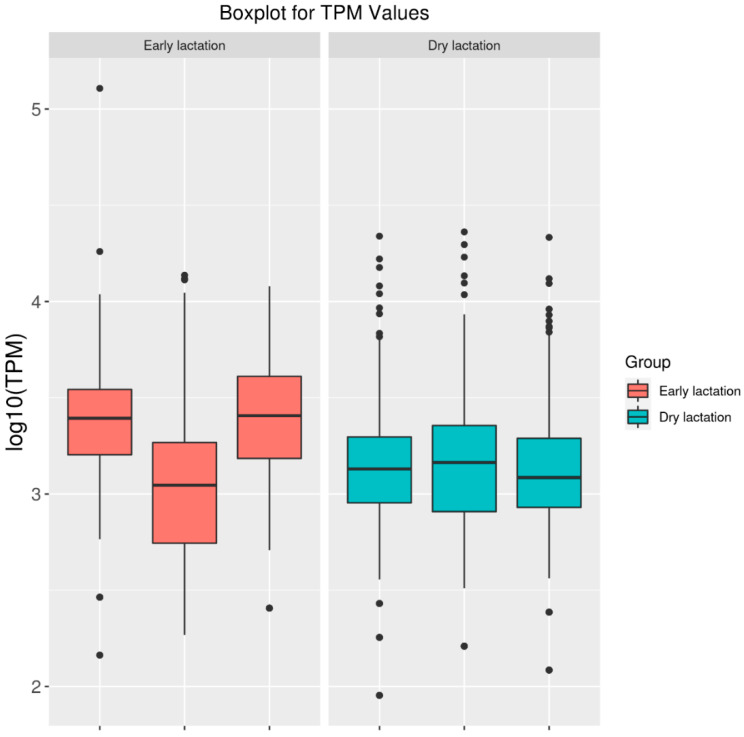
Boxplot diagram of the goat mammary gland during early lactation and dry lactation. TPM, transcripts per million.

Transcriptome sequencing results revealed 112,447,802–135,189,948 reads in the early lactation library and 124,554,188–127,449,174 reads in the dry/nonpregnant library (Table 1). The percentage of base recognition accuracy above 99.9% was 93.11% to 95.17% in the early lactation library and 93.28–94.93% in the dry/nonlactating library. The percentage of total mapped/clean reads was 92.11–97.20% in the early lactation library and 96.72–97.72% in the dry/nonpregnant library (Table 2). Furthermore, the percentage mapped to exons/mapped to genes was 37.12–42.83% in the early lactation library and 31.82–42.20% in the dry/nonlactating library. In addition, patterns of expression in both libraries were relatively uniform (Figure 1), indicating that the sequencing results could be used for subsequent research.

### 2.2. Differentially Expressed circRNAs between Stages of Lactation

**Figure 2:**
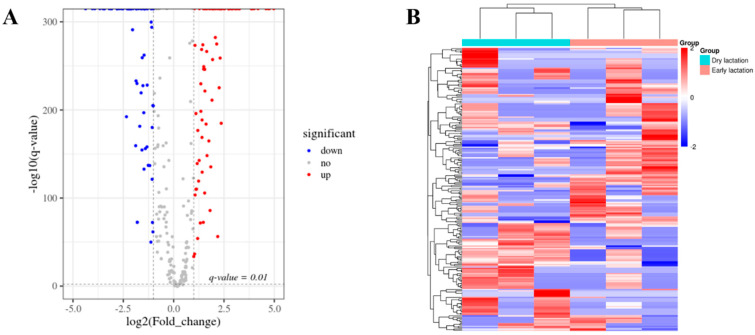
Differential expression of circRNAs in the goat mammary gland during early lactation and dry lactation. (**A**) Volcano plot of circRNAs expressed differently between early lactation and dry lactation; (**B**) heatmap of circRNAs expressed differently between early lactation and dry lactation.

As shown in Figure 2, the sequencing results revealed 215 differentially expressed circRNAs between the early and dry/nonpregnant stages. Compared to the dry/nonpregnant stage, 107 circRNAs were upregulated and 108 were downregulated in early lactation (Figure 2 and Table S1), allowing candidate circRNAs (including circ003429) to be studied (Table S2).

### 2.3. Enrichment Analysis

**Figure 3:**
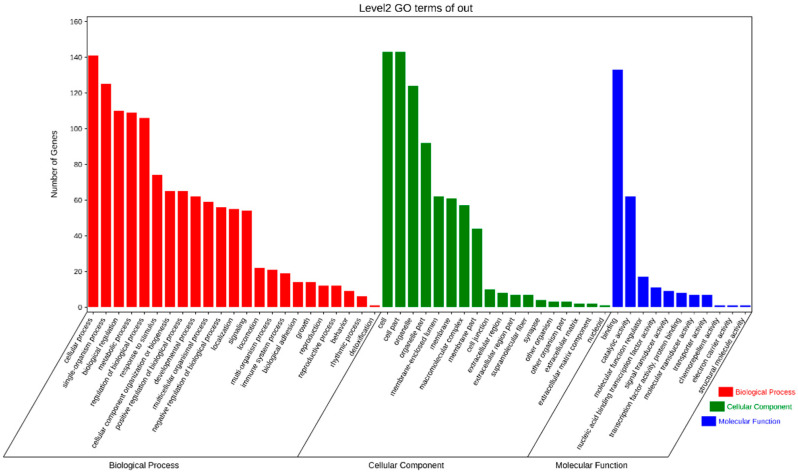
GO classification of differentially expressed genes.

**Figure 4:**
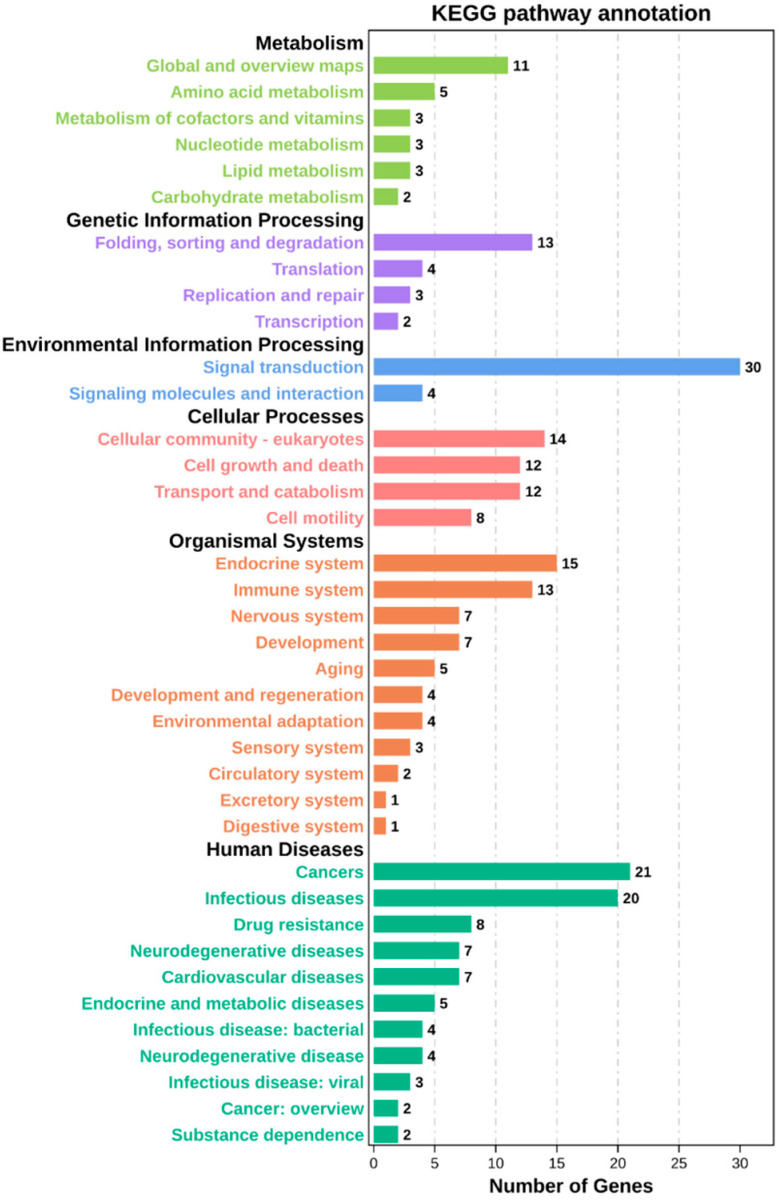
KEGG pathways of differentially expressed genes.

The GO enrichment analysis revealed 52 categories of differentially expressed circRNAs, including cellular components, cellular processes, binding, single-organism processes, organelles, biological regulation, and metabolic processes (Figure 3). The KEGG enrichment analysis revealed that the differentially expressed circRNAs were enriched in 184 pathways, including cancer, metabolic pathways, PI3K-Akt signaling pathway, regulation of actin cytoskeleton, focal adhesion, Alzheimer’s disease, human papillomavirus infection, and apoptosis (Figure 4). These results indicate that the circRNAs produced by these genes may play multiple roles in the mammary gland through these pathways.

### 2.4. Analysis of the Interaction between circRNA and miRNA

**Figure 5:**
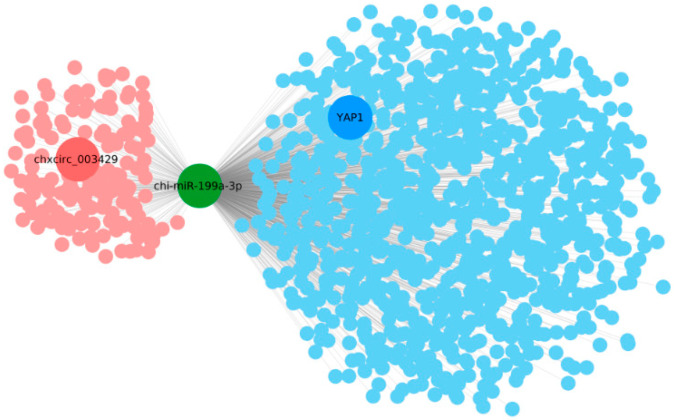
Circ003429-miR-199a-3p-*YAP1* interaction analysis.

CircRNAs can absorb, bind, and inhibit miRNA function. Thus, the miRNA target genes for the candidate circRNA were predicted to further explore their functions. In the present study, miRanda and Cytoscape 3.6.0 software was used to predict and identify circRNAs and mRNAs that targeted miR-199a-3p. The ceRNA network analysis indicated that circ003429 may target miR-199a-3p and that *YAP1* may be targeted by miR-199a-3p (Figure 5).

### 2.5. Circ003429 Adsorption and Binding of miR-199a-3p

**Figure 6:**
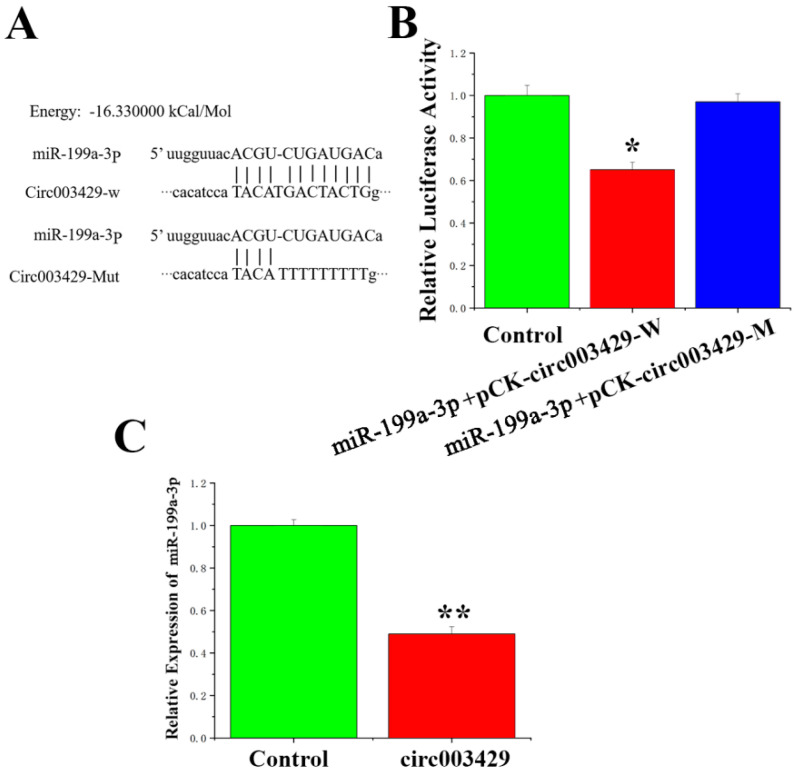
Analysis of the relationship between circ003429 and miR-199a-3p. (**A**) Sequence analysis of the binding site of miR-199a-3p and circ003429. (**B**) miR-199a-3p was cotransfected with pCK-circ003429-W or pCK-circ003429-Mut in 293T cells. (**C**) Effect of circ003429 on the expression levels of miR-199a-3p. Values are shown as the means ± standard errors. * *p* < 0.05; ** *p* < 0.01.

Sequencing results indicated that the exon start site and end site of circ003429 were 18,607,920 and 18,650,811, respectively, and originated on chromosome 27. In addition, a miR-199a-3p-binding site was identified in the circ003429 sequence (Figure 6A). To verify whether circ003429 binds miR-199a-3p, a wild-type recombinant vector mutant containing the miR-199a-3p binding site and a psicheck-2 vector with mutated binding sites was constructed (Table S3). The dual-luciferase reporter gene assay was used to identify whether miR-199a-3p has targeted binding ability. The results indicated that luciferase activity was reduced after cotransfection of miR-199a-3p and the wild-type vector. However, after cotransfection of miR-199a-3p and the mutant vector, luciferase activity was unchanged compared to the control group (Figure 6B). Furthermore, the expression of miR-199a-3p was reduced by the overexpression of circ003429 (Figure 6C). Therefore, these results indicated that circ003429 adsorbs and binds miR-199a-3p.

### 2.6. Functional Verification of circ003429 in GMECs

**Figure 7:**
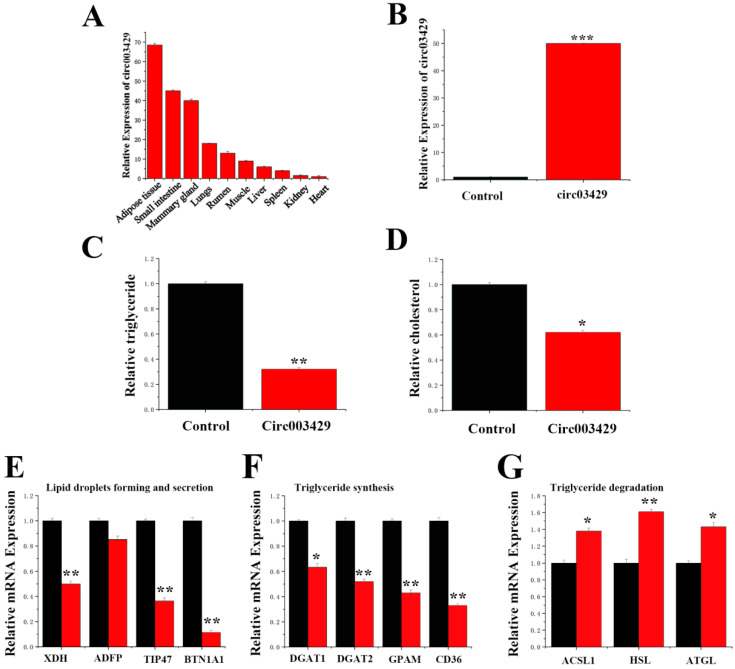
Functional verification of circ003429 in GMECs. (**A**) Tissue specific expression analysis of circ003429. (**B**) Expression efficiency of the circ003429 overexpression vector (pcDNA-circ003429). (**C**) Relative TAG levels. (**D**) Relative cholesterol levels. (**E**–**G**) Effects of circ003429 on genes related to lipid deposition and secretion in goat mammary epithelial cells. Black bars represent the negative control, and red bars represent circ003429. Values are shown as the means ± standard errors. * *p* < 0.05; ** *p* <0.01; *** *p* < 0.001.

We measured the expression level of circ003429 in different tissues of dairy goats (Figure 7A) and observed that circ003429 was primarily expressed in adipose, small intestine, and mammary tissues. Reverse primers were designed to detect circ003429. A circ003429 overexpression vector (pcDNA-circ003429) was constructed, and its function in GMECs was verified by qRT-PCR. The expression of circ003429 was increased by 49.8-fold after the overexpression vector was transfected into GMECs (Figure 7B), suggesting that this vector could be used to study the function of circ003429 in GMECs.

We detected the TAG and cholesterol levels in GMECs overexpressing circ003429, and the results revealed that the TAG and cholesterol levels were decreased (Figure 7C,D). In addition, circ003429 changed the composition of fatty acids by decreasing the contents of C16:1, C18:1, and C18:2 but increasing the contents of C16:0 and C18:0, leading to a greater fatty acid saturation index, which was also consistent with the decreased fatty acid accumulation in cells (Table 3).

As shown in Figure 7E–G, the levels of genes associated with lipid droplets formation and secretion (*XDH*, *TIP47* and *BTN1A1*) and triglyceride synthesis (*DGAT1*, *DGAT2*, *GPAM*, and *CD36*) were decreased, and the levels of genes associated with triglycerides degradation (*ACSL1*, *HSL* and *ATGL*) were increased in response to overexpression of circ003429. In addition, the level of *ADFP* was not significantly changed by circ003429. The decreased level of these genes was consistent with the lower TAG content induced by circ003429 overexpression.

### 2.7. MiR-199a-3p Specifically Targets YAP1 in GMECs

**Figure 8:**
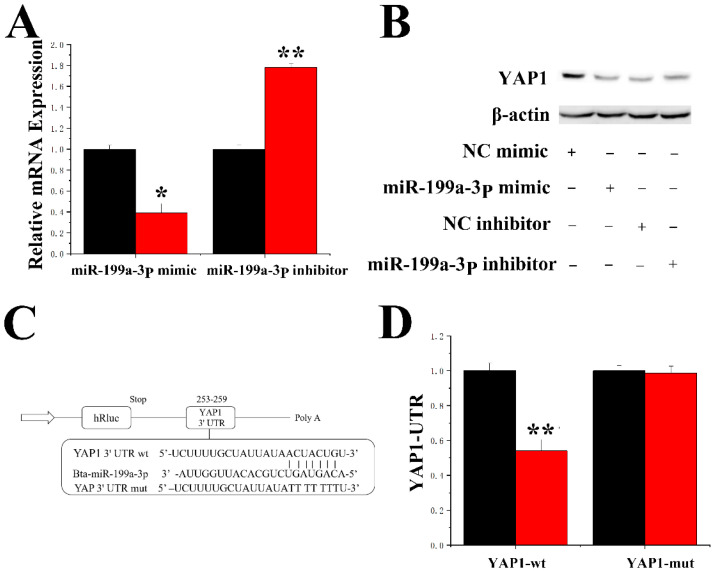
miR-199a-3p specifically targets *YAP1*. (**A**) mRNA expression levels of *YAP1* in response to the miR-199a-3p mimic and miR-199a-3p inhibitor. Black bars represent the negative control, and red bars represent the miR-199a-3p mimic or inhibitor. (**B**) Protein expression levels of *YAP1*. (**C**) Target site of miR-199a-3p in the *YAP1* 3′-UTR. (**D**) Construction of the luciferase (Luc) vector fused with the *YAP1* 3′-UTR. WT: Luc reporter vector with the WT *YAP1* 3′-UTR (253 to 259); MU: Luc reporter vector with a mutation at the miR-199a-3p site in the *YAP1* 3′-UTR. Values are presented as the means ± standard errors. * *p* < 0.05; ** *p* < 0.01.

**Figure 9:**
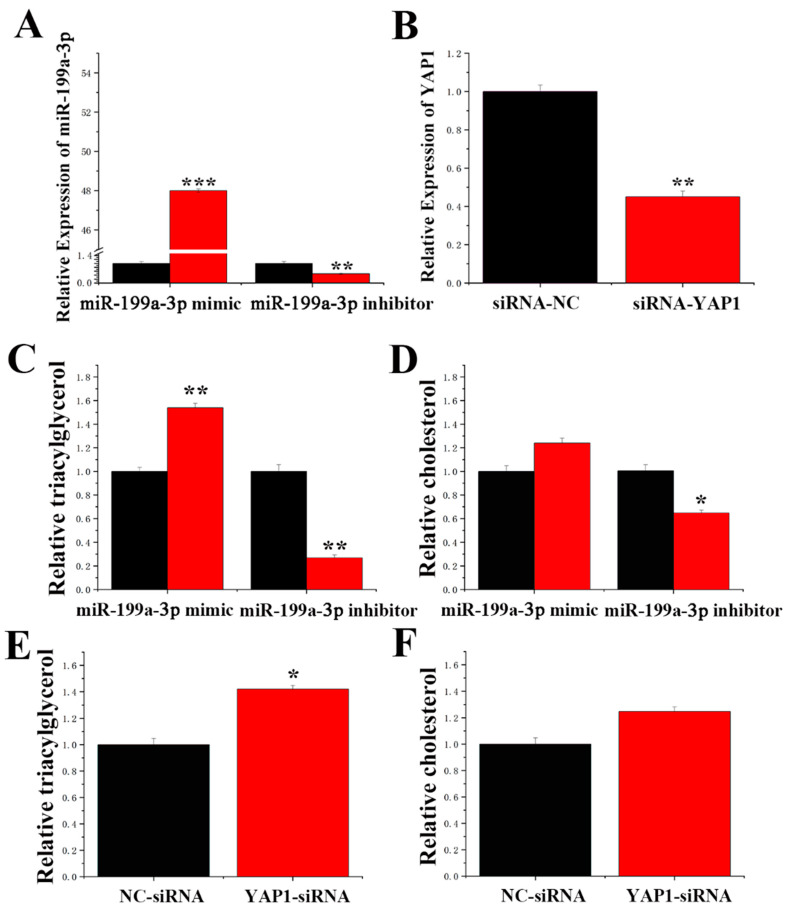
Functional evaluation of miR-199a-3p and *YAP1*. (**A**) Expression levels of miR-199a-3p. Black bars represent the negative control, and red bars represent the miR-199a-3p mimic or inhibitor. (**B**) mRNA expression levels of *YAP1*. (**C**) Relative TAG levels. (**D**) Relative cholesterol levels. Black bars represent the negative control, and red bars represent the miR-199a-3p mimic or inhibitor. (**E**) Relative TAG levels. (**F**) Relative cholesterol levels. Black bars represent the negative control, and red bars represent siRNA-*YAP1*. Values are shown as the means ± standard errors, * *p* < 0.05; ** *p* < 0.01; *** *p* < 0.001.

DAVID and TargetScan software predicted that miR-199a-3p bound the 3′-UTR of *YAP1*. *YAP1* expression was downregulated by the miR-199a-3p mimic and upregulated by the miR-199a-3p inhibition at the mRNA and protein levels (Figure 8A,B). To further confirm that miR-199a-3p directly targets *YAP1*, the 3′-UTR fragment containing the *YAP1* targeting site of miR-199a-3p was synthesized and cloned into the psi-check2 vector, and the 3′-UTR plasmid was constructed (Table S3). A reduction in luciferase activity of the wild-type reporter gene 3′-UTR was observed after miR-199a-3p overexpression, and the luciferase activity of the mutant reporter gene was not changed (Figure 8C,D). Moreover, as shown in Figure 9A, the expression levels of the miR-199a-3p mimic were 48-fold greater than that of the control group (NC-mimic, negative control mimic), and the expression levels after miR-199a-3p inhibitor treatment were reduced to less than 55% compared to the control group (NC-inhibitor, negative control inhibitor).

### 2.8. Function of miR-199a-3p and YAP1 in GMECs

As shown in Figure 9B, compared to the control group, the expression levels of siRNA-*YAP1* in GMECs were downregulated by more than 50% (NC-siRNA), indicating a high transfection efficiency of siRNA-*YAP1*. Compared to the control, the miR-199a-3p mimic increased TAG content by 50% (Figure 9C). In contrast, the TAG content was significantly decreased by inhibition of miR-199a-3p. Compared to the control, the cholesterol content was significantly decreased after treatment with the miR-199a-3p inhibitor (Figure 9D). The TAG content was increased by 40% in siRNA-*YAP1* GMECs compared to control GMECs (Figure 9E). However, cholesterol content was not changed in siRNA-*YAP1* GMECs compared to control GMECs (Figure 9F).

### 2.9. Circ003429 Combined with miR-199a-3p Relieves YAP1 Inhibition

**Figure 10:**
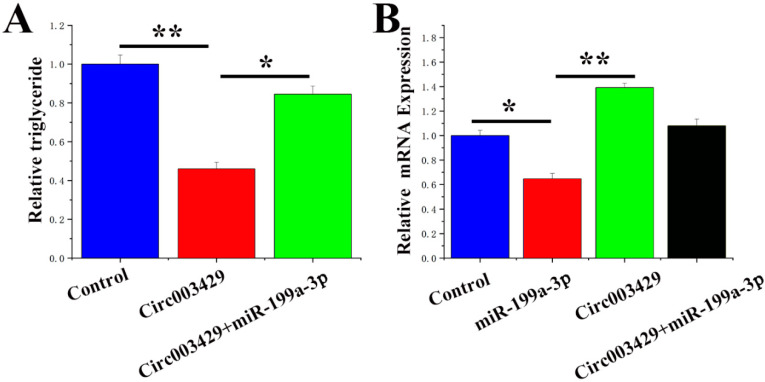
Circ003429 combined with miR-199a-3p relieves *YAP1* inhibition. (**A**) TAG levels in cells transfected with control, circ003429, or circ003429 + miR-199a-3p. (**B**) Circ003429 promotes mRNA expression of *YAP1*. *YAP1* expression levels in cells transfected with control, miR-199a-3p, circ003429 or circ003429 + miR-199a-3p. Values are presented as the means ± standard errors. * *p* < 0.05; ** *p* < 0.01.

A rescue experiment was conducted to examine the functional regulatory relationship between circ003429 and miR-199a-3p. Circ003429 decreased the TAG content in GMECs, and treatment with miR-199a-3p attenuated this effect (Figure 10A). In addition, the mRNA expression of *YAP1* was significantly upregulated by the overexpression of circ00342, and treatment with miR-199a-3p attenuated this effect (Figure 10B). Thus, these data indicated that circ003429 alleviates the inhibitory effect of miR-199a-3p on *YAP1*.

## 3. Discussion

The present study revealed mechanisms for competitive binding of circ003429 to miR-199a-3p in GMECs by overexpressing pcDNA-circ003429. Overexpression of miR-199a-3p increased TAG content and decreased mRNA abundance of *YAP1* (a target gene of miR-199a-3p), and TAG content was decreased and mRNA abundance was increased in response to overexpression of circ003429. Therefore, these data provide a new avenue for research and the theoretical basis for improving goat milk quality.

The wart gene encodes a Ser/Thr protein kinase in the Hippo signaling pathway and belongs to the NDR protein family [17]. Using genetic mosaic screening technology, Justice et al. [18] discovered that mutations in the wart gene (tumor suppressor gene) cause overgrowth in Drosophila, indicating a regulatory and control function on the size of tissues and organs. Further studies confirmed that the Hippo signaling pathway is conserved among species including mammals and that it primarily regulates organ size during development [19]. Regulation of the Hippo signaling pathway occurs through a series of kinase cascade reactions that cause the phosphorylation and inactivation of transcription factors to regulate downstream target genes [20,21]. Recently, research on the influence of the Hippo signaling pathway on lipid metabolism has attracted attention [22,23]. The Hippos signaling pathway plays an important regulatory role in the process of lipid development and differentiation [24]. By activating important signaling molecules upstream of the Hippo pathway, neurofibromatosis (neurofibromin 2, NF2) activity is reduced, causing a series of cascades of key factors in the Hippo pathway as follows: Mst1/2 are activated; Lats1/2 are phosphorylated and activated; and activated Latsl/2 further phosphorylates YAP [25]. Thus, studying the function and regulation of Lats2 is important in the context of Hippo signaling. Phosphorylation of YAP decreases its activity and anchors it in the cytoplasm. P-YAP and p-TAZ are locked and, thus, cannot enter the nucleus and bind to TEAD, causing TEAD to lose its transcriptional activity, which is equivalent to inhibiting the Hippo signaling pathway [26,27]. Under these conditions, the abundance of transcription factors involved in fat differentiation, such as *PPARG* and *C/EBPα*, is increased and enhances cell differentiation and lipid formation [22], suggesting that the Hippo signaling pathway is closely related to lipid metabolism. Previous studies have shown that *YAP1* inhibits adipogenic differentiation by inhibiting the transcriptional activity of PPARγ, a key factor in adipogenic formation [28]. PPARγ is a member of the nuclear hormone receptor superfamily that functions as a key transcription factor during adipogenesis. Overexpression of PPARγ also significantly upregulates the PPAR response element (PPRE) [29], which regulates the transcriptional activity of Stearoyl-Coenzyme A Desaturase 1 (*SCD1*) by directly binding to this element [30]. Overexpression of PPARγ in GEMCs results in upregulated *SCD1* expression, decreased contents of c16:0 and c18:0, and increased contents of c16:1 and C18:1 [31].

In the present study, siRNA was used as a knockout tool for *YAP1*. siRNA is a type of small RNA molecule with a unique biosynthetic mechanism from miRNA. Target gene mRNA is degraded by siRNA with precise sequence specificity. Previous studies have demonstrated that a small amount of siRNA strongly inhibited the abundance of target genes. It has been speculated that there is a doubling mechanism in the RNAi process, but this mechanism needs further experimental verification [32,33]. Thus, in this study, siRNA-*YAP1* was used to inhibit *YAP1* expression with a high degree of efficiency. To determine the competitive binding of circ003429 and miR-199a-3p to *YAP1*, a rescue experiment was designed. The results indicated that circ003429 inhibited TAG content. However, when circ003429 and miR-199a-3p were cotransfected into GMECs, the decrease in TAG content was alleviated. Furthermore, the expression level of *YAP1* was inhibited by miR-199a-3p and enhanced by circ003429. When circ003429 and miR-199a-3p were cotransfected into GMECs, the inhibition of *YAP1* was alleviated. Thus, circ003429 may mitigate the inhibitory effect of miR-199a-3p, which competitively binds *YAP1*, subsequently leading to changes in TAG.

The TAG content in cells is determined by the rate of synthesis and degradation. In the current study, overexpression of circ003429 significantly reduced the expression of genes related to TAG synthesis, lipid droplet formation, and secretion but increased the expression of genes related to TAG degradation, resulting in an overall reduction in TAG content in cells. Synthesis of TAG is achieved under the catalysis of *DGAT* activity [34]. In animals, *DGAT1* and *DGAT2* are encoded by different genes, but they have similar functions [35]. *DGAT1* is highly expressed in the small intestine and plays a significant role in the uptake of exogenous TAG [36,37]. In adipocytes, the absence of DGAT1 significantly reduces the synthesis of TAG [38]. Although *DGAT2* partly compensates for the insufficiency of TAG synthesis caused by the lack of *DGAT1* in the small intestine, the expression levels of *DGAT2* in the small intestine are not as high as those in the liver and white adipose tissue [37]. In mouse liver, deletion of *DGAT2* leads to severe TAG deficiency [39]. In the present study, overexpression of circ003429 reduced *DGAT* gene expression levels with a greater impact on *DGAT2*. Thus, we hypothesized that *DGAT2* plays a major role in TAG synthesis after circ003429 treatment, which was consistent with the report by Smith et al. [38], who speculated that *DGAT1* primarily uses exogenous fatty acids to synthesize TAG but that DGAT2 primarily uses endogenous fatty acids to synthesize TAG.

Circular RNAs are a newly identified class of closed-loop endogenous noncoding RNAs. CircRNAs were first discovered in 1976 by Kolakofsky [40] using electron microscopy in plant viruses and parainfluenza virus particles. In 1979, Hsu and Coca-Prados [41] discovered RNA in a circular structure in animal cells. In 1991, circRNA was first observed in human cells. However, due to the special structure of circRNAs and the limited research techniques at the time, only a few circRNAs were discovered in the subsequent 20 years [42]. More recently, the rapid development of high-throughput sequencing technology and bioinformatics analysis technology has provided an opportunity for circRNA research [43]. The mammary gland is an important organ for female mammals to feed their offspring. In addition to strict regulation by hormones, growth factors, and some proteins, circRNAs also play an important regulatory role in mammary development and lactation [44]. A previous study has identified 6824 and 4523 circRNAs in the rat mammary glands at two different stages of lactation, and a large number of circRNAs are specifically expressed at different stages of lactation [45]. Researchers have analyzed the expression profiles of circRNA in the mammary RNA library of dairy cows 90 and 250 days postpartum, identifying 4804 and 4048 circRNAs in the mammary glands at two stages of lactation, of which 2231 circRNAs are coexpressed, indicating that circRNA is highly specific to a stage of lactation [46].

The above studies suggest that circRNAs play an important role in the regulation of mammary function, but knowledge regarding the regulation of circRNAs during lactation remains limited. In the present study, we identified the miR-199a-3p-binding site in the circ003429 sequence, and we found that miR-199a-3p plays an important role in milk fat synthesis. Thus, we studied the function of circ003429 in GMECs. Dual-luciferase activity revealed that circ003429 displayed targeted binding with miR-199a-3p, and overexpression of the circ003429 (pcDNA-circ003429) sequence significantly inhibited the expression levels ofmiR-199a-3p. These findings revealed that the circ003429 sequence targets miR-199a-3p. Furthermore, research on the effects of circ003429 in cells demonstrated that circ003429 significantly inhibited TAG, which was contrary to the regulatory effect of miR-199a-3p overexpression in GMECs. Similarly, *YAP1* gene expression was significantly increased after overexpression of circ003429, which was also contrary to the inhibitory effect of miR-199a-3p on *YAP1*. Therefore, these results showed that circ003429 competitively binds to miR-199a-3p, which alleviates the inhibitory effect of miR-199a-3p on the *YAP1* target gene, thereby affecting milk fat metabolism.

## 4. Materials and Methods

### 4.1. Sample Collection

Three healthy dry/nonpregnant and three lactating Saanen dairy goats (Caremore, Daqing, China) were used to harvest mammary tissue (1–2 g) through surgical biopsy. Three 3-year-old goats of similar weight at early lactation (15 d after parturition) and the nonlactating period (i.e., “dry” period) were used. After the tissue samples were washed with DEPC water, they were stored in liquid nitrogen until RNA extraction. The animal care and surgery procedures of goats in the present study were performed according to the experimental license (protocol number: NEAU-(2011)-9) from Northeast Agricultural University (Harbin, China).

### 4.2. Sequencing Library Construction and High-Throughput Sequencing

TRIzol reagent (Invitrogen Life Technologies) was used to isolate total RNA. The quantity and quality of RNA was determined using a NanoDrop ND-1000 spectrophotometer (Nanodrop Technologies, Wilmington, DE, USA). RNA sequencing libraries were prepared using 2 μg of total RNA with some modifications. Ribosomal RNA was removed using an Epicenter Ribo-Zero™ rRNA Removal Kit (Human/Mouse/Rat, illumina, Madison, WI, USA), and linear RNA was removed using RNase R (Epicenter Biotechnologies, Madison, WI, USA). Poly-T oligo-attached magnetic beads were used to remove residual poly-A RNA. Fragmentation was performed using divalent cations under elevated temperature with an Illumina proprietary fragmentation buffer. First-strand cDNA was synthesized using RNA as a template and random oligonucleotides as primers. RNaseH was then used to degrade the RNA strand and in the DNA polymerase I system, dNTPs with dUTP were used instead of dTTP to synthesize second-strand cDNA. Double-stranded cDNA was purified, and double-end repair was performed followed by introduction of the “A” base at the 3′ end and connection to the sequencing adapter. Subsequently, USER enzyme (NEB, Beverly, MA, USA) was added to degrade the second strand of cDNA containing U. The AMPure XP system (Beckman Coulter, Beverly, CA, USA) was used to purify library fragments. A NovaSeq 6000 platform (Illumina, San Diego, CA, USA) was used to create the sequencing library (Personal Biotechnology Co. Ltd., Shanghai, China).

### 4.3. Acquisition of circRNAs

A 20 bp intercept from two ends of unmapped reads from the Tophat2 (2.0.14) (University of Maryland, College Park, MD, USA) alignment results was used to anchor reads. Bowtie2 (2.2.6) was then used to align these reads to the genome for circRNA detection. The alignment results from all samples were combined, and circRNAs were identified using find_circ (1.0). Highly credible circRNAs were then screened, and circRNAs were classified based on circRNA identification results and genome annotation information.

### 4.4. Expression Level Analysis

The fragments per kilobase of exon per million fragments mapped (FPKM) density distribution was used to examine the expression patterns of all circRNAs in each sample. In general, moderately expressed circRNAs comprised the majority with low- and highly expressed circRNAs accounting for a small portion. StringTie (v1.2.4) software was used to calculate FPKM and analyze the expression of circRNAs at the transcript level.

### 4.5. Analysis of Differential Expression of circRNAs

DESeq (1.30.0) was used to determine differentially expressed circRNAs. Transcripts with |log^2^ (fold change)| > 1 and q-value < 0.01 were considered differentially expressed.

### 4.6. Analysis of circRNA and miRNA Interactions

CircRNAs adsorb miRNAs and inhibit their function. Thus, the prediction of miRNA target genes in newly identified circRNAs facilitates the further investigation of circRNA function. MiRanda (v3.3a) and psRobot (1.2) software were used to predict miRNA target genes.

### 4.7. Quantitative Real-Time Polymerase Chain Reaction (RT–qPCR)

TRIzol kits (TaKaRa, Dalian, China) were used to extract total RNA from samples. Extracted RNA was treated with RNase R at 37 °C for 15 min, and the TaKaRa PrimeScript RT Kit (TaKaRa, Dalian, China) was used to synthesize cDNA. The general reverse transcription system included 500 ng of total RNA, 2 μL of 5 × Mix, 0.5 μL of Random6 primer, 0.5 μL of oligodT primer, and ddH_2_O to a final volume of 10 μL. The reverse transcription temperature program was 37 °C for 15 min, 85 °C for 5 s, and storage at 4 °C. The real-time fluorescent quantitative PCR instrument was a Bio-Rad CFX96 (Hercules, CA, USA). The reaction system volume was 25 μL, including 12.5 μL of SYBR Premix Ex Taq II, 10 ng of cDNA and 10 μmol/L upstream and downstream primers, and ddH_2_O was added to a final volume of 25 μL. The qRT–PCR (Thermo Fisher, Waltham, MA, USA) program was as follows: 95 °C for 30 s; 39 cycles of 95 °C for 5 s and 60 °C for 30 s; and 95 °C for 10 s and 65 °C for 5 s. Relative abundance was calculated using 2^−ΔΔCT^, and UXT was used as the internal control gene (Table S4).

### 4.8. Cell Culture

Approximately 1 g of mammary gland tissue was surgically collected from healthy and lactating goats (30 days), rinsed with buffer and then quickly separated. Resuspended cells were cultured at 37 °C, 5% CO_2_, and suitable humidity, and the medium was changed every 48 h. When cells reached approximately 80% confluence, 2 μg/mL prolactin was added to the basic medium to induce the formation and secretion of lipid droplets. The GMEC basal medium was Dulbecco’s modified Eagle medium (DMEM)/F12 supplemented with 10% fetal bovine serum, 5 μg/mL bovine insulin, and 10 kU/L green/streptomycin. Cells were transfected with either the miR-199a-3p mimic (60 nM) or inhibitor (60 nM) (Invitrogen, Waltham, MA, USA) using LipofectamineTM RNAMAX (Invitrogen, Waltham, MA, USA) according to the manufacturer’s instructions. Cells were harvested after 48 h of transfection. The sequences of the mimic, inhibitor, and siRNA are listed in Table S2.

### 4.9. Construction of circRNA Expression Vectors

The full-length circRNA sequence was amplified with HindIII and KpnI restriction endonuclease sites (Thermo, Beijing, China), and the bases were protected at both ends of the primers. The full-length sequence of circ003429 contained a loop-linking sequence connected to the pcDNA3.1 vector (Table S5).

### 4.10. Detection of TAG and Cholesterol Contents

The TAG and cholesterol contents were measured according to the methods described by Chen [47]. Briefly, cells were treated with lysis buffer and centrifuged after 48 h of incubation. Commercial kits (Loogen, Beijing, China) were used for the determination of TAG and cholesterol levels according to the manufacturer’s instructions. Protein assay kits (BCAs) (Thermo Fisher Scientific, Beijing, China) were used for protein quantification.

### 4.11. Western Blotting

The culture medium was discarded after treatment, and cells were washed 2–3 times with PBS. After the PBS was removed, 200 µL of RIPA lysis solution (containing 1:100 PMSF) was added to cells and incubated for 10 min. A vortex oscillator was used, and the cells were then collected and centrifuged at 12,000 rpm for 5 min followed by storage until use. Protein loading buffer was added to collected protein samples and placed in boiling water for 10 min to denature the proteins. To detect the abundance of *YAP1*, a *YAP1* rabbit anti-bovine polyclonal antibody was used as the primary antibody, and a goat anti-rabbit IgG-HRP antibody was used as the secondary antibody. To detect the abundance of the internal reference β-actin, a β-actin mouse anti-bovine monoclonal antibody was used as the primary antibody, and a goat anti-mouse IgG-HRP was used as the secondary antibody. BCA kits (Thermo, Beijing, China) were used for protein quantification.

### 4.12. Determination of Fatty Acid Content in GMECs

The content of fatty acids in GMECs was determined according to the method described by Chen [48]. In brief, 2 mL of 0.25% sulfuric acid methanol solution was added to 100 mg of cells, followed by ultrasonic disruption, and incubation at 80 °C for 1 h to methylate fatty acids. After the solution reached room temperature, 2 mL of 0.1 M HCL solution was added and mixed, and 800 µL of n-hexane was added followed by vortexing and shaking for 30 s. Samples were centrifuged at 900× *g* for 5 min at room temperature. The supernatant was transferred to a siliconized glass centrifuge tube, and approximately 0.5 g of anhydrous sodium sulfate was added prior to vortexing to remove water. After centrifugation at 13,800× *g* for 5 min at room temperature, the collected supernatant was used for GC-MS (Agilent CrossLab, Beijing, China) analysis of fatty acid composition and content.

### 4.13. Analysis of Dual-Luciferase Activity

In brief, 293A cells were seeded into a 48-well plate (7 × 10^4^ for each well), and the reporter gene plasmid vector was transfected using PEI reagents (Gobekie, Shanghai, China). After 4 h, the fluorescence was observed with a fluorescence microscope. After 48 h, the cells were washed 3 times with PBS, and then 40 μL of 1 × Passive Lysis Buffer was added followed by shaking for 15 min on a shaker. Lysates were collected in a 1.5 mL EP tube and centrifuged for 10 min at 3500 rpm. Supernatant (4 μL) was collected and mixed with 20 μL of LARII, and Lumat3 LB9508 was used to detect the fluorescence. Then, 20 μL of stop solution (Stop & Go) was added, and the internal reference fluorescence value was recorded. pcDNA-miR-199a-3p and recombinant psiCHECK-2-circ003429-W/psiCHECK-2-circ003429-Mut(pCK-circ003429-W/pCK-circ003429-Mut) vectors were cotransfected into HEK293T cells.

### 4.14. Statistical Analysis

Data were subjected to statistical analysis using one-way analysis of variance (ANOVA) with SPSS 18.0 software (SPSS, Chicago, IL, USA). The results are expressed as the means ± standard errors. Differences were considered significant at * *p* < 0.05; ** *p* < 0.01; *** *p* < 0.001.

## 5. Conclusions

**Figure 11:**
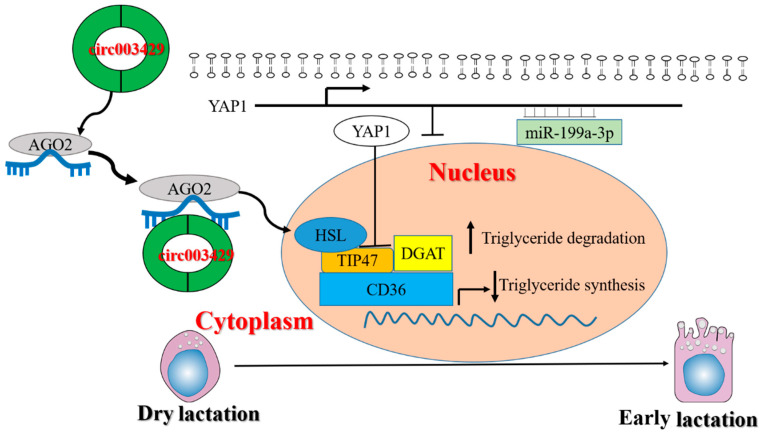
Circ003429 regulates unsaturated fatty acid synthesis in GMECs by adsorbing miR-199a-3p.

The regulatory mechanism of fatty acid metabolism in mammary glands involves the expression, network regulation, and signal transduction of multiple genes (including circRNAs). It has been found that the molecular mechanism of fatty acid metabolism regulation in the mammary gland is far more complex than we know, and there are still a considerable number of regulatory factors to be explored and identified. Circ003429 was identified by screening based on sequencing, and no study has been reported on the function of circ003429. Therefore, the present study systematically investigated the function of circ003429 in GMECs to clarify its function so as to improve the productivity of dairy goats and the quality of goat milk. In addition, after elucidating the regulatory effect of circ003429 on fatty acid synthesis, we further analyzed the regulatory mechanism of circ003429/miR-199a-3p/*YAP1* on fatty acid synthesis in GMECs and constructed the regulation network map of circ003429/miR-199a-3p/*YAP1*, which enriched the regulation theory of milk fat metabolism in goat mammary glands (Figure 11).

## Figures and Tables

**Table 1:** Sequencing Data Quality.

Samples	Read No.	Bases (bp)	Clean_Read No.	Clean Data (bp)	Q30	Q30 (%)
EL1	112,447,802	16,867,170,300	103,061,092	15,459,163,800	15,705,576,121	93.11
EL2	119,900,238	17,985,035,700	102,779,038	15,416,855,700	17,048,097,687	94.79
EL3	135,189,948	20,278,492,200	101,520,494	15,228,074,100	19,299,211,421	95.17
DL1	126,893,190	19,033,978,500	108,906,480	16,335,972,000	17,756,274,866	93.28
DL2	124,554,188	18,683,128,200	104,354,388	15,653,158,200	17,736,653,054	94.93
DL3	127,449,174	19,117,376,100	108,510,024	16,276,503,600	17,869,615,977	93.47

EL, early lactation; DL, dry lactation.

**Table 2:** Mapping statistics and events of RNASeq.

Item	EL1	EL2	EL3	DL1	DL2	DL3
Clean_Reads	103,061,092	102,779,038	101,520,494	108,906,480	104,354,388	108,510,024
Total_Mapped (%)	97.20	97.64	92.11	96.72	97.72	96.74
Multiple_Mapped (%)	2.09	1.92	3.12	4.13	1.54	3.69
Uniquely_Mapped (%)	97.91	98.08	96.88	95.87	98.46	96.31
Map_Events	98,081,017	98,425,684	90,599,563	100,986,515	100,406,806	101,099,167
Mapped_to_Gene (%)	73.50	72.42	76.07	74.13	72.81	74.46
Mapped_to_InterGene (%)	26.50	27.58	23.93	25.87	27.19	25.54
Mapped_to_Exon (%)	42.83	37.12	41.90	42.20	31.82	40.52

EL, early lactation; DL, dry lactation.

**Table 3:** Effects of circ003429 on fatty acids composition in GMECs.

Fatty Acid	NC	circ003429
C16:0 (%)	26.34 + 0.54	34.56 + 0.57 **
C16:1 (%)	18.34 + 0.65	5.47 + 1.038 **
C18:0 (%)	12.04 + 1.01	32.16 + 0.87 **
C18:1 (%)	34.71 + 1.54	25.36 + 1.67 *
C18:2 (%)	8.57 + 0.38	2.45 + 0.97 *
SFAs (%)	38.38	66.72
UFAs (%)	61.62	33.28
UFAs/SFAs	1.97	0.96

SFA, saturated fatty acids; UFA, unsaturated fatty acids. Values are presented as means ± standard errors. * *p* < 0.05; ** *p* < 0.01.

## Data Availability

Not applicable.

## Supplementary Materials

The following supporting information can be downloaded at: https://www.mdpi.com/article/10.3390/ijms23074068/s1.
